# Assessment of Genetic Diversity and Differentiation in *Triadica cochinchinensis* Populations Using SSR Markers

**DOI:** 10.3390/plants15081209

**Published:** 2026-04-15

**Authors:** Pengyan Zhou, Qi Zhou, Chenghao Zhang, Meng Xu, Yingang Li

**Affiliations:** 1 Institute of Tree Breeding, Zhejiang Academy of Forestry, 399 Liuhe Road, Hangzhou 310023, China; pyzhou@njfu.edu.cn (P.Z.); qizhou36@hotmail.com (Q.Z.); 2 Zhejiang Key Laboratory of Forest Genetics and Breeding, Zhejiang Academy of Forestry, 399 Liuhe Road, Hangzhou 310023, China; 3 Co-Innovation Center for Sustainable Forestry in Southern China, Nanjing Forestry University, Nanjing 210037, China; zchh18270856918@163.com (C.Z.); xum@njfu.edu.cn (M.X.); 4 State Key Laboratory of Tree Genetics and Breeding, College of Forestry, Nanjing Forestry University, Nanjing 210037, China

**Keywords:** *Triadica cochinchinensis*, SSR markers, genetic diversity, genetic differentiation

## Abstract

Genetic diversity is fundamental for the conservation and sustainable utilization of plant species. *Triadica cochinchinensis*, a tree species native to southern China, is an important ornamental and nectar-producing plant with considerable economic value. However, the levels of genetic diversity and the patterns of population differentiation across its natural populations remain unexplored. Here, we developed 24 highly polymorphic SSR markers and used them to assess the genetic diversity and differentiation among 280 individuals collected from 10 natural populations of *T. cochinchinensis*. The results showed that the average expected heterozygosity (He) revealed by the SSR markers was 0.774, and the average Shannon diversity index (I) was 1.660, indicating a high level of genetic diversity at the species level of *T. cochinchinensis*. Analysis using SSR markers revealed a low average observed heterozygosity (Ho = 0.323) and a relatively high average inbreeding coefficient within populations (F = 0.466). These findings suggest that inbreeding is likely occurring, which may contribute to a loss of heterozygosity within the studied populations. Notably, not all populations had high genetic diversity. For example, the He of SC2 population (0.490), QY population (0.568), and SC1 population (0.585) were all below the mean He (0.607), suggesting that attention should be given to protecting populations with low genetic diversity. The results further showed that the average genetic differentiation coefficient (F_ST_) between populations was 0.094, and the average gene flow (Nm) was 2.278, indicating that the natural populations of *T. cochinchinensis* had low genetic differentiation and relatively high gene flow. AMOVA indicated that 74% of the total variation was distributed within populations. Notably, populations SC1 and SC2 exhibited higher genetic differentiation from all others (F_ST_ > 0.1), which is likely attributed to mountain barriers restricting gene flow. Therefore, it is recommended to enhance in situ conservation efforts while also facilitating assisted gene flow, such as through artificial introduction. For the first time, this study reveals the genetic information of natural populations of *T. cochinchinensis* at the molecular level, thereby offering a valuable reference for the conservation and utilization of its germplasm resources.

## 1. Introduction

*Triadica cochinchinensis* Loureiro (Euphorbiaceae), commonly referred to as the red-leafed tallow tree, is a broad-leaved species native to southern China. It belongs to the genus *Triadica*, and its natural range spans southern provinces including Zhejiang, Hunan, Jiangxi, and Fujian, where it typically occurs at elevations between 200 and 1600 m. *T. cochinchinensis* prefers deep, well-drained moist soils, exhibits considerable drought tolerance, and demonstrates strong resistance to pests and diseases, contributing to its broad environmental adaptability. Valued for its vivid foliage, rapid growth, ease of cultivation, and extended ornamental period, it is commonly used as a specimen tree in landscaping or as a primary species for establishing seasonal red-leaf forests [1]. Furthermore, *T. cochinchinensis* has a prolonged flowering period of 30–40 days, produces abundant inflorescences, and secretes large amounts of nectar, ranking it among the major summer nectar sources in southern China. The honey derived from it is nutritionally rich, with notably higher iron and zinc content compared to acacia honey or jujube honey, and is associated with benefits such as anemia improvement and immune enhancement. Additionally, its seeds contain up to 20% oil, rendering them suitable for soap and candle production [2]. As a species that offers ecological, economic, and social benefits, *T. cochinchinensis* has broad potential for promotion and application. However, existing research on *T. cochinchinensis* had primarily focused on apiculture, phenotypic characterization, phylogenetic studies, silviculture and afforestation [3,4,5,6]. In contrast, systematic studies on the genetic diversity and population differentiation of its natural populations remain unexplored, thus limiting the informed selection and breeding of superior germplasm.

At present, the survival and evolutionary potential of plant populations are increasingly threatened by the combined effects of anthropogenic disturbance and environmental stress. Genetic diversity, a cornerstone of biodiversity, is crucial for maintaining population health, safeguarding adaptive resilience, and supporting the sustainable use of plant genetic resources [7]. Consequently, the assessment of genetic diversity is a prerequisite for the effective conservation and utilization of plant genetic resources. With advances in molecular biology, the methods for such assessment have gradually progressed from morphological and cytological approaches to the analysis of DNA variation. Molecular markers, capable of detecting DNA-level variation and accurately identifying genotypes, are key instruments for analyzing genetic diversity, population structure, and gene flow [8,9]. Various marker types are available, including ISSR, AFLP, SSR, and RFLP. In previous studies, PCR amplification of ISSR markers had been studied in *T. cochinchinensis* [3], but the authors did not investigate its population genetic diversity and differentiation. Due to the high polymorphism and stability, SSR (simple sequence repeat) markers are widely applied in population genetic diversity analysis, genetic structure analysis, and conservation genetics in plants. For example, Wang et al. used SSR markers to study the genetic structure of the important protected tree species *Phoebe sheareri* and predicted the impacts of climate change on *P. Sheareri* [10]; Zhou et al. employed SSR markers to conduct genetic research on the secondary protected plant *Liriodendron chinense*, finding a low level of genetic diversity and high genetic differentiation [11]; Zhang et al. used SSR markers to study *Juglans mandshurica* populations and found a high level of genetic diversity and moderate genetic differentiation [12]. These studies are important for understanding the genetic diversity and genetic structure of species, allowing researchers to propose corresponding conservation strategies or potential utilization prospects for different populations of the species. However, there have been no reports on the application of SSR markers to the study of genetic diversity in *T. cochinchinensis* populations.

Here, we report the first population genetic study of *T. cochinchinensis* based on 280 samples from 10 natural populations using 24 SSR markers. Our objectives were to: (1) characterize genetic diversity at both the species and population levels, and (2) evaluate the patterns of gene flow and genetic differentiation among natural populations of *T. cochinchinensis*. These results advance the understanding of genetic diversity and genetic structure in *T. cochinchinensis*, offering essential insights for the management and conservation of its natural populations and the exploitation of its germplasm.

## 2. Results

### 2.1. Polymorphism of SSR Markers

From 100 initially designed SSR primers, 85 (85%) successfully amplified clear fragments of the expected size (150–400 bp) across six test samples, while 15 (15%) failed to produce any PCR product. Based on polymorphism and amplification stability, 24 primer pairs were selected from the successful set for subsequent analysis in *T. cochinchinensis* (Table S1).

Null allele frequencies estimated by CERVUS ranged from 0.003 to 0.184 across the 24 loci, with a mean of 0.080 (Table 1). Simultaneously, the mean of the proportions of significant linkage disequilibrium (LD) pairs was 0.76% (Table S2). The results showed that all 24 SSR markers met the requirements for further diversity and structure analyses.

A total of 233 alleles were detected at the 24 SSR loci across all 280 individuals from 10 populations (Table 1). The average observed number of alleles (Na) per locus was 9.708, with individual locus values ranging from 4 (marker SWJ095) to 23 (marker SWJ044). Marker SWJ044 displayed the following maximum values: effective number of alleles (Ne = 16.397), expected heterozygosity (He = 0.941), and polymorphism information content (PIC = 0.936). Additionally, marker SWJ098 and marker SWJ027 also indicated relatively high genetic diversity. Specifically, marker SWJ098 exhibited the highest observed heterozygosity (Ho = 0.743) coupled with a high He (0.887). Marker SWJ027 showed high values for both He (0.880) and Ne (8.238) compared to most other markers. In contrast, several markers revealed lower diversity: marker SWJ004 had the lowest Ne (2.547) and He (0.609); marker SWJ046 showed the lowest PIC (0.531), a low Ne (2.555), and a relatively low He (0.610); and marker SWJ040 displayed relatively low values for both Ne (2.618) and He (0.619).

Overall, the average expected heterozygosity (He) and Shannon’s diversity index (I) across all loci were 0.774 and 1.660, respectively, collectively indicating a high species-level of genetic diversity in *T. cochinchinensis*. However, it is worth noting that the average Ho revealed across all loci was only 0.323, indicating a low level of observed heterozygosity and a deficiency of heterozygotes within the population.

### 2.2. Genetic Diversity

Based on the genetic diversity indices (Table 2), the He among the 10 populations ranged from 0.490 to 0.671, while Ho varied from 0.207 to 0.418. The SR population exhibited the highest He (0.671) and Ho (0.418), along with the second-highest I (1.326). The YH1 population showed the highest Ne (3.477) and I (1.328), as well as the second-highest He (0.667). Together, these metrics indicated that the SR and YH1 populations harbored the highest genetic diversity in this study. In addition, the YP population displayed the highest allelic richness (AR = 5.568) and Na (5.667), coupled with a relatively high He (0.638). It was notable that the JO population displayed the second-highest AR (5.432) and a relatively high He (0.638). YP and JO populations also had the highest number of private alleles (5), which suggested that both YP and JO populations not only possessed high genetic diversity but might also contain distinctive allelic variation. Conversely, the SC2 population showed the lowest values for He (0.490), Ne (2.400), and I (0.906). The QY population followed, with He = 0.568, Ne = 2.696, and I = 1.071, indicating comparatively reduced diversity. Notably, although the He of the SC1 population was relatively low (0.585), this population harbored the highest number of private alleles (5), suggesting that the SC1 population contained unique genetic resources.

Overall, *T. cochinchinensis* maintained a relatively high population-level genetic diversity (mean He = 0.607) and possessed abundant allelic resources (mean AR = 4.917). However, the Ho within populations was relatively low (average Ho = 0.324), and the average inbreeding coefficient within populations was relatively high (F = 0.466), suggesting that inbreeding may be occurring within populations, thereby contributing to a loss of heterozygosity.

### 2.3. Genetic Differentiation and Genetic Structure

Pairwise genetic differentiation (F_ST_) values among populations are presented in Table 3. The overall mean F_ST_ was 0.094, accompanied by an average gene flow (Nm) of 2.278 (Table S3). Notably, the highest differentiation was observed between SC2 and WYS (F_ST_ = 0.231), followed by SC1 and WYS (0.173), SC2 and SR (0.170). Overall, populations SC1 and SC2 showed greater differentiation from the other populations (F_ST_ > 0.1). This pattern may be attributed to the geographic barriers posed by the Xianxia and Wuyi Mountains, which likely restrict gene flow between the SC populations and the remaining populations. In contrast, genetic differentiation was lowest between JO and YP (F_ST_ = 0.045), and second lowest between QY and JN (F_ST_ = 0.058), indicating relatively frequent gene exchange among these populations.

The results of the AMOVA revealed that 9% of the total genetic variation resided among regions, 17% of the total genetic variation occurred among populations, and 74% of the total variation was distributed within the populations (Table 4). This suggests that variation among individuals within the population constitutes the primary component of genetic variation in *T. cochinchinensis*.

**Figure 1:**
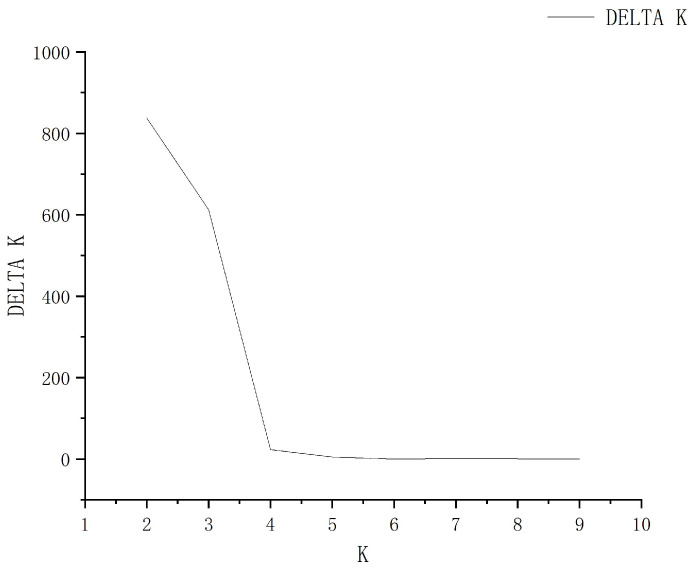
Evaluation of the optimal number of genetic clusters (K) in *T. cochinchinensis* based on the ΔK statistic from STRUCTURE.

**Figure 2:**
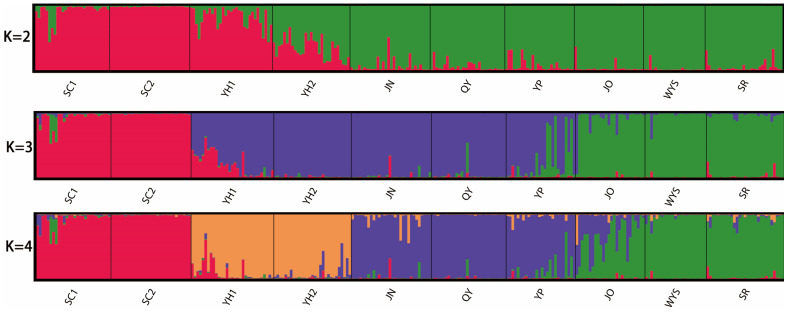
Bayesian clustering results for *T. cochinchinensis* populations at K = 2–4, illustrating the inferred genetic structure. For K = 2, the red cluster comprises SC1, SC2, and YH1; the green cluster comprises JN, QY, YP, JO, WYS, and SR; and YH2 showed an admixture of red and green. For K = 3, the red cluster comprises SC1 and SC2, the green cluster comprises JO, WYS, and SR, and the purple cluster comprises YH1, YH2, JN, QY, and YP. For K = 4, the red cluster comprises SC1, SC2, the orange cluster comprises YH1, YH2, the purple cluster comprises JN, QY, YP, and the green cluster comprises JO, WYS, SR.

To infer the population genetic structure of *T. cochinchinensis*, we employed a Bayesian analysis using STRUCTURE. The optimal number of genetic clusters was determined to be K = 2 based on the highest ΔK value (Figure 1), dividing the ten populations into two main groups (Figure 2). For K = 2, populations SC1, SC2, and YH1 were predominantly assigned to one ancestral cluster (shown in red), while JN, QY, YP, JO, WYS, and SR largely derived from the other (green). Population YH2 showed an admixed ancestry. For K = 3, SC1 and SC2 formed a distinct cluster; JO, WYS, and SR clustered together; and YH1, YH2, JN, QY, and YP shared a third common ancestry. The pattern at K = 4 further resolved the structure, separating the groups as follows: (i) SC1 and SC2, (ii) YH1 and YH2, (iii) JN, QY, and YP, and (iv) JO, WYS, and SR.

**Figure 3:**
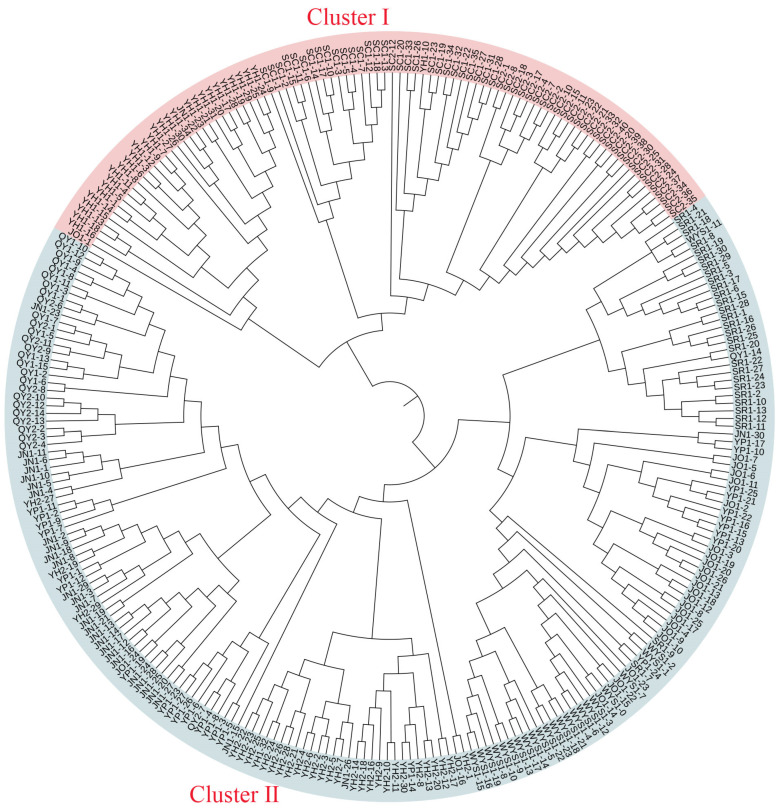
UPGMA tree of *T. cochinchinensis* based on Nei’s genetic distances.

**Figure 4:**
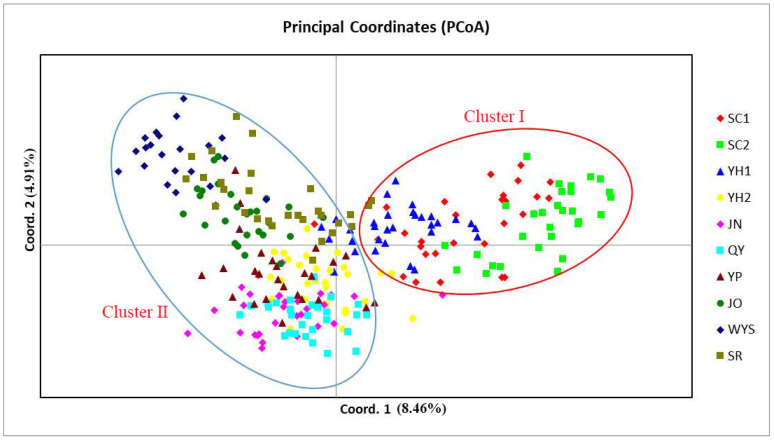
Principal coordinate analysis (PCoA) of the 10 *T. cochinchinensis* populations.

Notably, the UPGMA analysis (Figure 3) and PCoA (Figure 4) yielded closely congruent patterns, consistently identifying two distinct genetic clusters: Cluster I comprised SC1, SC2, and YH1, while all other populations formed Cluster II. Overall, these results indicated a relatively simple genetic architecture in *T. cochinchinensis*.

## 3. Discussion

Genetic diversity serves as a critical foundation for the conservation of forest germplasm resources and for efficient breeding programs. It encompasses the extent of variation within populations, thereby providing the essential genetic material for developing new cultivars with desirable traits such as high yield, stress tolerance, and improved quality [13,14]. Moreover, assessing and studying genetic diversity not only advances our understanding of species’ population structure and gene flow patterns, but also supplies a vital evidence base for developing scientifically informed strategies for the conservation and sustainable utilization of genetic resources.

SSR markers are a well-established tool for assessing genetic diversity and constructing genetic maps in plants. Their utility was evidenced by numerous studies: Hu et al. used 18 SSR markers to reveal low genetic diversity (He = 0.470) in the endangered *Saussurea involucrata*, thereby informing its conservation [15]. Conversely, studies on garlic (*Allium sativum*) by Li et al. (29 SSR markers, He = 0.670) and *Akebia trifoliata* by Chen et al. (72 SSR markers, He = 0.720) reported relatively high diversity, aiding germplasm utilization and breeding [16,17]. Notably, within the same family (Euphorbiaceae), Zhou et al. found low diversity (He = 0.491) in *T. sebifera*, highlighting conservation needs for its natural populations [18]. In this study, we developed 24 polymorphic SSR markers for *T. cochinchinensis* and applied them to assess the genetic diversity of ten natural populations. Notably, loci such as SWJ044, SWJ098, and SWJ027 (all with He > 0.880) were identified as highly informative markers, thereby offering efficient tools for future genetic studies and germplasm characterization of *T. cochinchinensis*. The results revealed a relatively high species-level of genetic diversity (mean He = 0.774), which exceeded that reported for several other species mentioned previously. The genetic diversity of plant species is jointly influenced by their biological characteristics and ecological processes [19]. The efficient animal pollination mechanism of *T. cochinchinensis* may be one of the key factors maintaining its relatively high genetic diversity. The high expected heterozygosity (He = 0.774) indicates that *T. cochinchinensis* populations contain abundant allelic variation, providing important genetic variation for the species’ long-term evolutionary adaptation [20]. However, the present study revealed a relatively low level of observed heterozygosity (Ho = 0.323) in *T. cochinchinensis*, which was associated with a high inbreeding coefficient at the population level (F = 0.466). The reproductive characteristics of *T. cochinchinensis* can explain this inbreeding occurrence. First, *T. cochinchinensis* is a monoecious tree species that predominantly outcrosses. It produces numerous inflorescences over a long flowering period, with a large number of male and female flowers often present simultaneously on the same individual. This creates natural opportunities for pollen transfer between different flowers of the same plant, thereby leading to inbreeding. Second, although the oil-rich seeds of *T. cochinchinensis* are consumed by various bird species and thus possess some potential for bird-mediated dispersal, available evidence indicates that a large proportion of seeds still disperse mainly by gravity, resulting in relatively limited dispersal distances. Consequently, biparental inbreeding may occur, increasing the proportion of homozygotes, reducing observed heterozygosity, and causing observed heterozygosity to be lower than expected heterozygosity (Ho < He).

Based on genetic structure, UPGMA clustering, and PCoA analyses, the ten populations of *T. cochinchinensis* were delineated into two distinct genetic clusters. Populations SC1, SC2, and YH1, situated in the Xianxia mountains, comprised cluster I, while all remaining populations formed cluster II. When individuals from these genetically differentiated clusters were pooled, the overall heterozygosity of the mixed sample could be systematically underestimated, which further reduced observed Ho [21]. Therefore, the heterozygote deficiency observed in *T. cochinchinensis* likely results from the combined effects of inbreeding within populations and genetic structure among them.

Elucidating population differentiation and genetic structure is essential for understanding the spatial distribution of genetic diversity, which directly informs conservation and breeding strategies. This differentiation is primarily driven by natural selection, while gene flow acts as a counteracting force that homogenizes populations [22,23]. Studies using SSR markers report a range of scenarios, from high differentiation and low gene flow in *Camellia chekiangoleosa* (F_ST_ = 0.239, Nm = 0.796) [24] to low differentiation and high gene flow in *Ginkgo biloba* (F_ST_ = 0.093, Nm = 2.427) [25]. Our study placed *T. cochinchinensis* closer to the latter pattern, with an overall low genetic differentiation (F_ST_ = 0.094) and relatively high gene flow (Nm = 2.278) among populations. Nevertheless, significant genetic differentiation was detected among specific populations. Genetic structure analysis revealed that populations SC1 and SC2 possessed highly consistent genetic composition and showed substantial differentiation from populations in Cluster II (pairwise F_ST_ generally > 0.1). The divergence was most pronounced between SC1/SC2 and population WYS (mean F_ST_ = 0.231), likely attributable to the barrier posed by the Xianxia and Wuyi mountain ranges, which restricted gene flow and thereby enhanced genetic differentiation.

Concurrently, the Suichang populations (SC1 and SC2) exhibited relatively low levels of genetic diversity (mean He = 0.538, mean Ho = 0.241). This pattern was likely attributable to two interacting factors: first, a high inbreeding coefficient within these populations (F = 0.530) potentially induced inbreeding depression, where homozygous exposure of deleterious alleles reduces individual fitness; second, their distinct genetic structure limited effective gene flow, rendering them more vulnerable to the cumulative effects of genetic drift and inbreeding [26,27]. However, it is worth noting that the SC1 population had the highest number of private alleles (5). Consequently, the unique genetic resources of the SC1 population should be protected, and measures should be taken to enhance gene flow.

In contrast, YH1 of cluster I exhibited higher diversity (He = 0.667), likely due to genetic admixture with Cluster II. Cluster II, encompassing all remaining populations, displayed relatively higher overall diversity (mean He = 0.617). Within this cluster, SR, YP, and JO populations harbored the highest diversity (mean He = 0.649), whereas QY and WYS showed lower levels (mean He = 0.574). Conservation strategies should thus be tailored to these inter- and intra-cluster genetic patterns.

Therefore, *T. cochinchinensis* possesses substantial genetic diversity at the species level but exhibits signs of inbreeding depression within local populations. While its high expected heterozygosity represented a valuable evolutionary resource, the significantly reduced observed heterozygosity and elevated inbreeding coefficient underscore the need for active genetic management. Effective conservation must therefore aim not only to preserve extant populations but also to mitigate inbreeding, facilitate gene flow, and ultimately translate latent genetic variation into sustained adaptive capacity. Accordingly, conservation strategies should prioritize measures that enhance heterozygosity, thereby improving the long-term evolutionary potential and resilience of *T. cochinchinensis* populations.

In summary, this study offered a critical scientific foundation and actionable guidelines for the conservation of natural populations and genetic resource management of *T. cochinchinensis*. Accordingly, we propose a tiered conservation strategy tailored to distinct population genetic profiles. At the species level, priority should be given to core populations with high genetic diversity (e.g., SR, YP, JO), which should serve as key reserves for germplasm collection and breeding programs. For populations exhibiting signs of inbreeding depression (e.g., SC1, SC2), in situ conservation must be reinforced and coupled with active management, such as assisted gene flow through controlled pollination with genetically distinct individuals from Cluster II, to enhance heterozygosity. At the landscape level, conservation planning should address genetic differentiation caused by geographic barriers like the Xianxia Mountains. Efforts to facilitate gene exchange between genetic clusters (e.g., Cluster I and II) are essential to maintain the species’ evolutionary potential and adaptive resilience. Overall, by revealing the high species-level genetic diversity (He = 0.774) and population structure, this research provided a reference for designing targeted conservation and sustainable utilization strategies for *T. cochinchinensis*.

## 4. Materials and Methods

### 4.1. Plant Material and DNA Extraction

**Figure 5:**
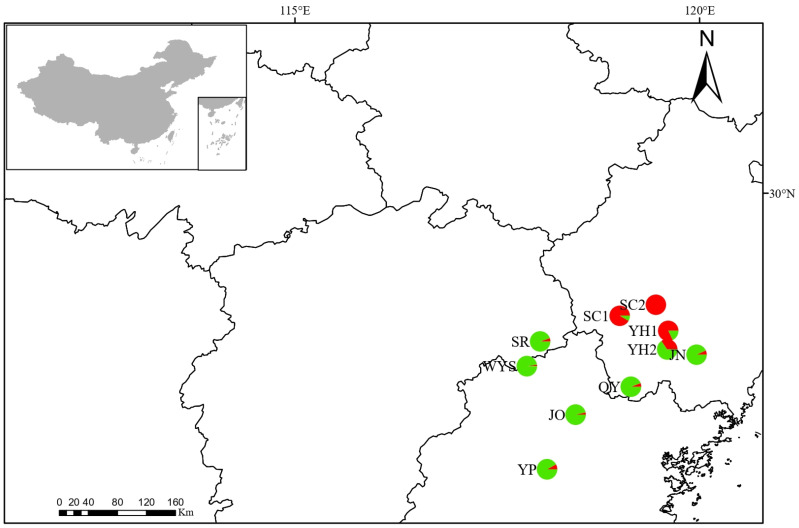
Sampling locations of the ten *T. cochinchinensis* populations. Pie charts on the map represent the predominant genetic ancestry of each population, categorized into two clusters (red and green) as inferred from the STRUCTURE analysis (K = 2).

In this study, a total of 280 individuals of *T. cochinchinensis* were collected from 10 natural populations across Zhejiang, Fujian and Jiangxi provinces of China (Figure 5, Table 5). Among them, populations from Zhejiang Province are predominantly encircled by the Xianxia Mountains, while those from Jiangxi and Fujian Provinces are largely surrounded by the Wuyi Mountains. The maximum elevation of the Xianxia Mountains is 1500 m, and the elevation of the Wuyi Mountains varies between 1000 and 2100 m. The *T. cochinchinensis* populations examined in this study were sampled from elevations ranging from 400 to 1500 m. To minimize the collection of closely related individuals and ensure genetic independence, a minimum distance of 50 m was maintained between sampled trees within each population. Fresh leaves were collected into dry sample collection bags, immediately placed on dry ice to rapidly halt enzymatic degradation, and subsequently stored at −80 °C for long-term preservation of genomic integrity.

Total genomic DNA of *T. cochinchinensis* was extracted using Super Plant Genomic DNA Kit (Tiangen Biotech, Beijing, China) following the manufacturer’s protocol. The concentration of DNA samples was detected by NanoDrop 2000c (ThermoFisher Scientific, Waltham, MA, USA). DNA quality was further verified by 1% agarose gel electrophoresis to confirm high molecular weight and the absence of significant degradation. Qualified DNA was diluted to 50 ng/µL for polymerase chain reaction (PCR) and stored at 4 °C.

### 4.2. SSR Primers Development and PCR Amplification

One hundred primer pairs were designed using Primer Premier 5.0 based on the genomic sequences of *T. cochinchinensis*, and screened using six randomly selected individuals from the total individuals (N = 280). A total of 24 SSR primer pairs exhibiting high polymorphism were retained for further genetic analysis (Figure S1, Table S1). PCR amplifications were carried out in a 25 μL volume comprising 12.5 μL of 2 × T5 Super PCR Mix, 1 μL of each primer (10 μM), 1 μL of genomic DNA (50 ng/μL), and 9.5 μL of ddH_2_O. The PCR programme was as follows: 98 °C for 3 min; 30 cycles of 98 °C for 10 s, 56 °C for 10 s, and 72 °C for 10 s; and a final extension of 72 °C for 1 min. The amplification products were separated and detected on a Qsep100 Q-Analyzer (Bioptic, Beijing, China). Genotyping results were read by Peak Scanner version 1.0 software (Applied Biosystems, Waltham, MA, USA) [28].

### 4.3. Data Analysis

The presence of null alleles was assessed using CERVUS v3.0 [29]. Loci with F(null) > 0.20 are typically considered problematic [30]. Linkage disequilibrium (LD) was tested for each population using Arlequin v3.5 with 10,000 permutations [31]. The exact *p*-value from the permutation test was used. Bonferroni correction for multiple comparisons was applied within each population (α = 0.00018). Only locus pairs with corrected *p* < 0.00018 were considered to show significant LD.

Genetic diversity analysis was performed using POPGENE v1.32 to estimate key parameters, including the observed number of alleles (Na), effective number of alleles (Ne), observed heterozygosity (Ho), and expected heterozygosity (He)—each providing insight into the distribution and richness of genetic variation within and among populations. PowerMarker v3.25 was employed to calculate the polymorphism information content (PIC), a measure of the informativeness of each marker [32]. Allelic richness (AR), which standardizes allele counts across different sample sizes, was estimated using FSTAT version 2.9.3 [33]. To visualize genetic relationships, a phylogenetic tree was constructed in MEGA v11.0.13 based on genetic distance, applying the Unweighted Pair-Group Method with Arithmetic Mean (UPGMA) clustering algorithm [34].

Population differentiation and spatial genetic patterns were examined through multiple complementary approaches. GenAlEx v6.5 was used to conduct an analysis of molecular variance (AMOVA), partitioning genetic variation within and among populations, and principal coordinate analysis (PCoA) for visualizing genetic distances among individuals and populations. The same software was also used to calculate genetic differentiation (F_ST_) and gene flow (Nm) [35]. To infer population genetic structure without a priori population designations, a Bayesian clustering analysis was implemented in STRUCTURE v2.3.1 [36]. The analysis assumed an admixture model with correlated allele frequencies. For each value of K (the number of putative ancestral populations, ranging from 1 to 11), we ran a Markov Chain Monte Carlo (MCMC) simulation with 1,000,000 iterations after a burn-in period of 1,000,000 iterations to estimate individual ancestries under an admixture model. The optimal K value was identified using the STRUCTURE HARVESTER online platform (http://taylor0.biology.ucla.edu/structureHarvester/) (accessed on 9 March 2025) [37]. Finally, to obtain consistent cluster assignments across runs, results were integrated using CLUMPP and graphically displayed with DISTRUCT software [38,39].

## 5. Conclusions

This study provides the first report on the population genetics of *T. cochinchinensis* using a novel set of 24 highly polymorphic SSR markers across ten natural populations. At the species level, *T. cochinchinensis* possesses relatively high genetic diversity (mean He = 0.774), which exceeded that reported for species such as *S. involucrata*, *T. sebifera* and certain other woody plants. However, analysis using SSR markers revealed a low average observed heterozygosity (Ho = 0.323) and a relatively high average inbreeding coefficient within populations (F = 0.466). These results suggest the likely occurrence of inbreeding, which may be contributing to a loss of heterozygosity in the studied populations. In addition, significant variation exists among populations. For instance, the SC2 population showed notably lower diversity (He = 0.490), highlighting it as a priority for genetic conservation. Overall, the populations exhibited low-to-moderate genetic differentiation (mean F_ST_ = 0.094) and considerable gene flow (mean Nm = 2.278). A notable exception was the significant differentiation observed for the Suichang populations (e.g., F_ST_ between SC2 and WYS = 0.231), suggesting potential isolation. For these populations, facilitated gene flow through managed introduction could be a future conservation strategy. These findings revealed the molecular-level genetic information of *T. cochinchinensis*, thereby offering a critical reference for its germplasm utilization and species conservation.

## Figures and Tables

**Table 1:** Polymorphism information of 24 SSR loci.

Locus	Na	Ne	Ho	He	PIC	I	F(null)
SWJ004	12	2.547	0.339	0.609	0.575	1.334	0.061
SWJ012	7	3.073	0.161	0.676	0.624	1.328	0.068
SWJ017	9	5.399	0.186	0.816	0.793	1.870	0.098
SWJ019	11	5.253	0.511	0.811	0.784	1.839	0.043
SWJ023	6	4.701	0.336	0.789	0.756	1.651	0.093
SWJ024	17	7.086	0.236	0.860	0.846	2.293	0.170
SWJ026	11	5.528	0.271	0.821	0.796	1.925	0.157
SWJ027	14	8.238	0.446	0.880	0.867	2.280	0.121
SWJ031	8	3.242	0.168	0.693	0.644	1.380	0.115
SWJ040	6	2.618	0.246	0.619	0.566	1.183	0.048
SWJ044	23	16.397	0.239	0.941	0.936	2.907	0.078
SWJ046	9	2.555	0.461	0.610	0.531	1.160	0.019
SWJ059	17	7.928	0.386	0.875	0.861	2.267	0.088
SWJ063	7	4.324	0.111	0.770	0.732	1.590	0.184
SWJ068	7	2.838	0.479	0.649	0.612	1.350	0.028
SWJ076	6	3.162	0.089	0.685	0.629	1.321	0.149
SWJ082	8	3.271	0.204	0.696	0.650	1.444	0.112
SWJ083	6	3.158	0.450	0.685	0.628	1.324	0.004
SWJ287	5	2.669	0.407	0.626	0.565	1.170	0.029
SWJ090	5	2.953	0.479	0.663	0.595	1.176	0.013
SWJ092	10	3.579	0.214	0.722	0.689	1.623	0.121
SWJ093	10	4.713	0.079	0.789	0.759	1.756	0.100
SWJ095	4	3.258	0.518	0.694	0.639	1.272	0.027
SWJ098	15	8.746	0.743	0.887	0.876	2.384	0.003
Mean	9.708	4.885	0.323	0.774	0.706	1.660	0.080

Na: observed number of alleles, Ne: effective number of alleles, He: expected heterozygosity, Ho: observed heterozygosity, PIC: polymorphism information content; I: Shannon’s index; F(null): null allele frequency estimate.

**Table 2:** Genetic diversity indices for 10 *T. cochinchinensis* populations based on 24 SSR loci.

Population	Na	Ne	I	Ho	He	AR	F	Number of Private Alleles
SC1	4.917	2.855	1.135	0.275	0.585	4.787	0.532	5
SC2	3.833	2.400	0.906	0.207	0.490	3.747	0.527	1
YH1	5.583	3.477	1.328	0.335	0.667	5.423	0.484	4
YH2	4.958	3.302	1.198	0.303	0.629	4.802	0.534	2
JN	4.833	3.212	1.176	0.329	0.605	4.721	0.464	2
QY	4.458	2.696	1.071	0.320	0.568	4.379	0.446	1
YP	5.667	3.391	1.294	0.361	0.638	5.568	0.435	5
JO	5.542	3.357	1.275	0.354	0.638	5.432	0.439	5
WYS	5.000	2.986	1.148	0.342	0.579	5.000	0.410	3
SR	5.458	3.460	1.326	0.418	0.671	5.310	0.395	1
Mean	5.025	3.113	1.078	0.324	0.607	4.917	0.466	29

F: inbreeding coefficient, AR: allele richness.

**Table 3:** Matrix of pairwise genetic differentiation (F_ST_) among *T. cochinchinensis* populations.

	SC1	SC2	YH1	YH2	JN	QY	YP	JO	WYS	SR
SC1	0.000									
SC2	0.103	0.000								
YH1	0.098	0.116	0.000							
YH2	0.120	0.147	0.063	0.000						
JN	0.129	0.169	0.097	0.062	0.000					
QY	0.127	0.159	0.103	0.081	0.058	0.000				
YP	0.107	0.155	0.092	0.073	0.064	0.060	0.000			
JO	0.137	0.169	0.108	0.095	0.095	0.091	0.045	0.000		
WYS	0.173	0.231	0.139	0.126	0.124	0.144	0.104	0.074	0.000	
SR	0.125	0.170	0.095	0.095	0.099	0.097	0.086	0.085	0.096	0.000

**Table 4:** Analysis of molecular variance of *T. cochinchinensis*.

Source	*d.f.*	Sum of Square	Mean of Square	Variance Components	Percentage of Variation
Among Regions	1	323.900	323.900	1.736	9%
Among populations	8	873.923	109.240	3.395	17%
Within populations	270	3961.534	14.672	14.672	74%
Total	279	5159.357		19.803	100%

**Table 5:** Sampling information for the *T. cochinchinensis* populations.

Population Code	Sample Size	Location	Longitude (°E)	Latitude (°N)
SC1	28	Suichang1, Zhejiang	119.01	28.48
SC2	30	Suichang2, Zhejiang	119.46	28.62
YH1	31	Yunhe1, Zhejiang	119.61	28.30
YH2	29	Yunhe2, Zhejiang	119.60	28.07
JN	30	Jingning, Zhejiang	119.96	28.00
QY	28	Qingyuan, Zhejiang	119.15	27.61
YP	26	Yanping, Fujian	118.12	26.58
JO	26	Jian’ou, Fujian	118.47	27.26
WYS	23	Wuyishan, Fujian	117.86	27.86
SR	29	Shangrao, Jiangxi	118.03	28.17
Total	280			

## Data Availability

The original contributions presented in this study are included in the article/Supplementary Material. Further inquiries can be directed to the corresponding author.

## Supplementary Materials

The following supporting information can be downloaded at: https://www.mdpi.com/article/10.3390/plants15081209/s1, Figure S1. Results of PCR product separation for partial loci; Table S1. Characteristics of 24 SSR markers developed for *Triadica cochinchinensis*; Table S2. Summary of significant linkage disequilibrium (LD) pairs across ten populations after Bonferroni correction; Table S3. Pairwise gene flow between *T. cochinchinensis* populations.
