# Supplementary material for: Assessment of Genetic Diversity and Differentiation in Triadica cochinchinensis Populations Using SSR Markers
Source: Plants (Basel). 2026 Apr 15;15(8):1209. doi: 10.3390/plants15081209 (PMC13119846; doi:10.3390/plants15081209)
Supplement: Supplementary file 1 [file plants-15-01209-s001.zip › plants-4142771-supplementary.pdf]

Supplementary Materials

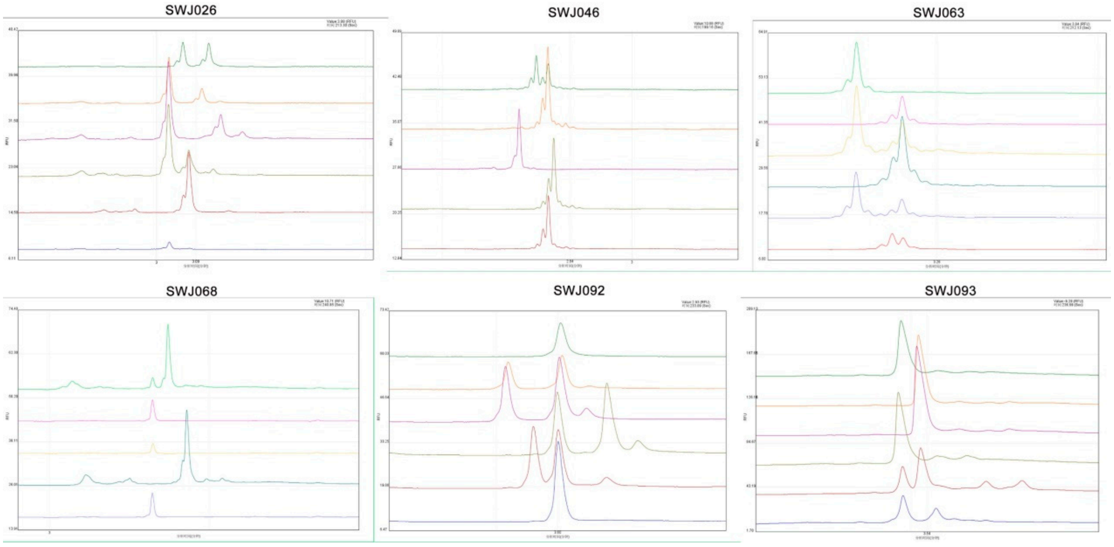

Figure S1. Results of PCR product separation for partial loci.

Table S1. Characteristics of 24 SSR markers developed for *Triadica cochinchinensis*

Locus	Repeat Motif		Primer sequence	Tm（℃）	Fragment size（bp）
SWJ004	(TA)7(TG)7	F	TTTCAAAAGGAAATCCGTGC	59	262
		R	ACAGTCCCTGGATGTCTTGG		
SWJ012	(A)10(TAT)9	F	TGAGTTTGGGTGTTGGCATA	59	284
		R	TGGTTCATACCCCCATCTTC		
SWJ017	(TAT)6(T)11	F	GCCAAGATGTCTGCCTTCAT	60	295
		R	TGGGTAAGGGTGAGCAAAAC		
SWJ019	(GAA)33	F	ACAGTAACATTAGCTCTGCTCAAC	57	285
		R	CGCTATGTCATCGGAGGATT		
SWJ023	(TTC)5tttttcttcttctt(CTC)7	F	ACCCCATACAACCATCTCCA	60	239
		R	CCCATGAACCTAACCGATTG		
SWJ024	(TAT)13tatccca(TAT)11	F	AAATCTACTCAACCACATTAACTACCA	58	261
		R	ACACAACGACAAAGTCAGCG		
SWJ026	(TAT)6ttacacctg(ATC)11	F	GAGCCTCAGCTACGCACACT	60	293
		R	CATCTCTACACATTCTTCCACCA		
SWJ027	(CTG)5(CTT)10	F	GGGAAAGTTGGGATGGTTTT	60	213
		R	ACCCTAATTACAGGGTCGGG		
SWJ031	(GAG)5(AAG)9	F	TAATGATGGGCCTTTCAAGC	60	299
		R	TTTCCCCTCCATTCTCTTCA		
SWJ040	(TTTC)6(TTC)8	F	GGTGCAAGAGCAGGACATTT	60	214
		R	TCCTGGAGGAAGGCACATAC		
SWJ044	(TAT)13	F	GCAGTTTTCCATGAGAGAATCA	59	287

		R	TGCCACTGCCTAATTTGATG		
SWJ046	(TAA)11	F	TGCACTTTAATGGATGATGGG	60	175
		R	GGGGATGTGGGAATTAGGTT		
SWJ059	(TAA)14(TTA)7	F	CGTCCAGCTGATATGCGTTA	59	251
		R	AGCAAGCCATAAAGCACCAC		
SWJ063	(TAT)12	F	ACCAATGAGCTGTTCCCATC	59	198
		R	AATCGTTGCTCCACTTCACC		
SWJ068	(ATAGAG)6	F	AACTCAAACGCCTCACGACT	59	229
		R	AGCAAAAGCAAGGCGAAATA		
SWJ076	(AATAT)5	F	AAATACCTGTCTCACGCGCT	59	297
		R	CTTTTGCTTCACGTGCGATA		
SWJ082	(TTA)14	F	TTGGCTTCCGGATGAAATTA	60	296
		R	CTCCCTCTTCCCTTCTGTCC		
SWJ083	(CATCAC)6	F	GCTTCGCTAAAGACGCAAAC	60	215
		R	CCTCGACTACAAGGCAAAGG		
SWJ087	(ACTCA)7	F	AGGATCCACAGTCCACAACC	59	226
		R	AGGCAAAATCAGGTTTGAGC		
SWJ090	(AAAGA)7	F	TTGGCCTACTCCTTCTTCACA	59	228
		R	GAAGGTGAAAATGGCTTTGC		
SWJ092	(TAA)11	F	AGCTCACAGACCGAAAGTGG	60	251
		R	CAAAGGGCATAAAAACGGAA		
SWJ093	(TAA)5(GAA)8	F	GGTCACTTAACCCCTCGTGA	59	274
		R	AGTCGTTTAACCTCTCGCCA		
SWJ095	(TCCCCG)5	F	GATCCGACACGAAGACCAAT	59	260
		R	TTAAAAAGCCCAACACCCTG		
SWJ098	(ATT)13	F	GCCAACATGATGCCATTTTA	59	269
		R	TTAAAGCTAACCCAAACGACA		

Table S2. Summary of significant linkage disequilibrium (LD) pairs across ten populations after Bonferroni correction

Population	Number of significant LD pairs	Percentage of total comparisons (%)
SC1	0	0
SC2	7	2.54
YH1	2	0.72
WYS	3	1.09
SR	0	0
YH2	2	0.72
YP	1	0.36
JN	2	0.72
QY	1	0.36
JO	3	1.09
Total / Mean	21	0.76

Table S3. Pairwise gene flow between *T. cochinchinensis* populations

	SC1	SC2	YH1	YH2	JN	QY	YP	JO	WYS	SR
SC1	0.000									
SC2	2.177	0.000								
YH1	2.301	1.905	0.000							
YH2	1.833	1.451	3.718	0.000						
JN	1.688	1.229	2.327	3.782	0.000					
QY	1.719	1.322	2.177	2.836	4.060	0.000				
YP	2.086	1.363	2.467	3.175	3.656	3.917	0.000			
JO	1.575	1.229	2.065	2.382	2.382	2.497	5.306	0.000		
WYS	1.195	0.832	1.549	1.734	1.766	1.486	2.154	3.128	0.000	
SR	1.750	1.221	2.382	2.382	2.275	2.327	2.657	2.691	2.354	0.000

Nm: gene flow
